# Supplementary material for: Neurobiological successor features for spatial navigation
Source: Hippocampus. 2020 Jun 25;30(12):1347–55. doi: 10.1002/hipo.23246 (PMC8432165; doi:10.1002/hipo.23246)
Supplement: Supplementary file 1 — AppendixS1: Supporting information [file HIPO-30-1347-s002.docx]

**Supplementary methods**

All simulations were implemented in MATLAB 2018b using the same set of 160 BVCs with parameters $\sigma_{ang}=11.25$, $\beta=12$, $\xi=8$. We used 16 preferred angles (0, 22.5, 45, 67.5, 90, 112.5, 135, 157.5, 180, 202.5, 225, 247.5, 270, 292.5, 315, 337.5) at 10 preferred distances (3.3cm, 10.2cm, 17.5cm, 25.3cm, 33.7cm, 42.6cm, 52.2cm, 62.4cm, 73.3cm, 85.0cm) chosen to provide uniform overlap between consecutive angular and radial tunings. Environments were discretised into 1x1cm bins for analysis, with rate maps filtered by a 13x13 Gaussian smoothing kernel with $\sigma=3$cm. All simulations learned the SR $\tilde{M}$ by implementing equation 8 at a rate of 50Hz with $\alpha_{\tilde{M}}=1x{10}^{-4}$ and $\gamma= 0.995$. Unless otherwise stated (such as in Figure 4), learning of $\tilde{M}$ was implemented using 2 hours of random foraging trajectory simulated using a model that mimics rodent foraging (Raudies & Hasselmo, 2012).

The environment dimensions used for the simulations in Figure 3 were 60x60cm, 60x120cm, 120x60cm and 120x120cm. The simulations for Figure 4 used a 65x65cm box with the insertion of a 40cm barrier. The environments used in Figure 5 consisted of a 95x95cm box, an 80cm diameter circle, and a trapezoid formed of base lengths of 20cm and 90cm with height 172cm. ­

Gridness scores were calculated following (Hafting, Fyhn, Molden, Moser, & Moser, 2005), with gridness thresholds used to identify the eigenvectors for analysis in Figures 5c-f identified using the 95th percentile of a field shuffling procedure (Barry & Burgess, 2017). The orientations of the main 3 axes of a ‘grid cell’ were identified using the central part of the spatial autocorrelogram encompassing the central peak and the 6 closest surrounding peaks. The orientations were calculated as the angles between the horizontal central axis and the 3 lines connecting the 3 closest peaks to the central peak in an anticlockwise order. Due to the symmetry in spatial autocorrelograms, the other 3 peaks have the same orientation modulo 180°. To assess the orientation clustering of grid-cells in the square and circular environments in Figure 5ef, we calculated the Kullback-Leibler (KL) divergence between the distributions of the grid orientations and a uniform distribution using a bin width of 12°.

The KL divergence $D_{KL}$ is a measure of how different a probability distribution $P$ (grid orientations) is from a reference distribution $Q$ (uniform distribution) defined on the same probability space.

$$D_{KL}(P||Q)= \sum_{x} P\left( x \right)\log\frac{P(x)}{Q(x)}$$

$D_{KL}\geq0$ and $D_{KL}=0$ if and only if $P=Q$.

The Bayes Factor reported compares the likelihood of the grid orientations in the circle as a result of two models – one model being that they share the same the distribution of orientations as in the square environment, and another being that they are sampled from a uniform distribution of orientations.

For comparison with the standard SR model, the environment was discretised into 1x1cm bins and the SR matrix $M$ was computed as the discounted sum of one-step transition matrices $T$ describing a uniform random walk on this state-space: $M= \sum_{t=0} \gamma^{t}T^{t}$ (Stachenfeld, Botvinick, & Gershman, 2017). As with the BVC-SR model, a discount factor of $\gamma= 0.995$ was used. The ellipticity and firing rate variability analyses were computed for the 95x95cm square environment.

The grid cell rate map ellipticity reported for the biological data (Krupic, Bauza, Burton, Barry, & O’Keefe, 2015) was calculated by fitting an ellipse to the 6 peaks surrounding the central peak of the spatial autocorrelogram. The eccentricity *e* of this ellipse used as the measure of the grid ellipticity:

$$e= \sqrt{1-\frac{b^{2}}{a^{2}}}$$

where a and b are the lengths of the longer and shorter axis of the fitted ellipse. Since neither the BVC-SR or standard SR models yield exclusively hexagonal grid patterns, ellipticity of the eigenvector rate maps was calculated by thresholding the spatial autocorrelogram at a value of 0.2 and identifying the central peak. Then the eccentricity *e* of this central peak was used as the measure of the grid ellipticity,where a and b are the lengths of the longer and shorter axis of the central peak.

Firing rate variability of the eigenvector rate maps was analysed following the method of Ismakov et al., (2017). Grid fields were identified using the watershed transform of each eigenvector rate map, and the coefficient of variability (CV) was calculated as the standard deviation of these peaks, divided by the mean of the peaks.

**References**

Barry, C., & Burgess, N. (2017). To be a Grid Cell: Shuffling procedures for determining “Gridness.” *BioRxiv*, 230250. https://doi.org/10.1101/230250

Hafting, T., Fyhn, M., Molden, S., Moser, M.-B., & Moser, E. I. (2005). Microstructure of a spatial map in the entorhinal cortex. *Nature*, *436*(7052), 801–806. https://doi.org/10.1038/nature03721

Ismakov, R., Barak, O., Jeffery, K., & Derdikman, D. (2017). Grid Cells Encode Local Positional Information. *Current Biology*, *27*(15), 2337-2343.e3. https://doi.org/10.1016/j.cub.2017.06.034

Krupic, J., Bauza, M., Burton, S., Barry, C., & O’Keefe, J. (2015). Grid cell symmetry is shaped by environmental geometry. *Nature*, *518*(7538), 232–235. https://doi.org/10.1038/nature14153

Raudies, F., & Hasselmo, M. E. (2012). Modeling Boundary Vector Cell Firing Given Optic Flow as a Cue. *PLoS Comput Biol*, *8*(6), 1002553. https://doi.org/10.1371/journal.pcbi.1002553

Stachenfeld, K. L., Botvinick, M. M., & Gershman, S. J. (2017). The hippocampus as a predictive map. *Nature Neuroscience*. https://doi.org/10.1038/nn.4650

**Supplementary Figure 1:** Grid fields generated using eigenvectors from the BVC-SR model are less elliptic than those from the standard SR model. Lower values indicate more circular fields and larger values indicate more elliptic fields, with a value of 0 indicating a perfect circle. a) Grid fields generated using the BVC-SR model had significantly lower ellipticity than the standard SR model (mean field ellipticity ± SD: 0.59 ± 0.23 vs. 0.75 ± 0.25; t(318)=-5.93; p<0.001), and were similar to observations of real grid cells (Krupic, Bauza, Burton, Barry, & O’Keefe, 2015). b) Histogram of the grid field ellipticity (N=160 eigenvectors)

**Supplementary Figure 2:** Grid fields generated using eigenvectors from the BVC-SR model exhibit more firing rate variability than the standard SR model. Following the method of Ismakov et al., (2017), the peak firing rates of grid fields was used to compute a coefficient of variability for each eigenvector (CV; SD divided by mean). a) The CV for eigenvectors produced by the BVC-SR model were significantly larger than that observed in the standard SR model (mean CV ± SD: 0.48 ± 0.11 vs 0.14 ± 0.11; t(318)=26.5; p<0.001), and similar to that observed in real grid cells (Ismakov et al., 2017). b) Histogram of the CV for each of the models (N=160 eigenvectors).
