# Supplementary figures and images for: Neurobiological successor features for spatial navigation
Source: Hippocampus. 2020 Jun 25;30(12):1347–55. doi: 10.1002/hipo.23246 (PMC8432165; doi:10.1002/hipo.23246)

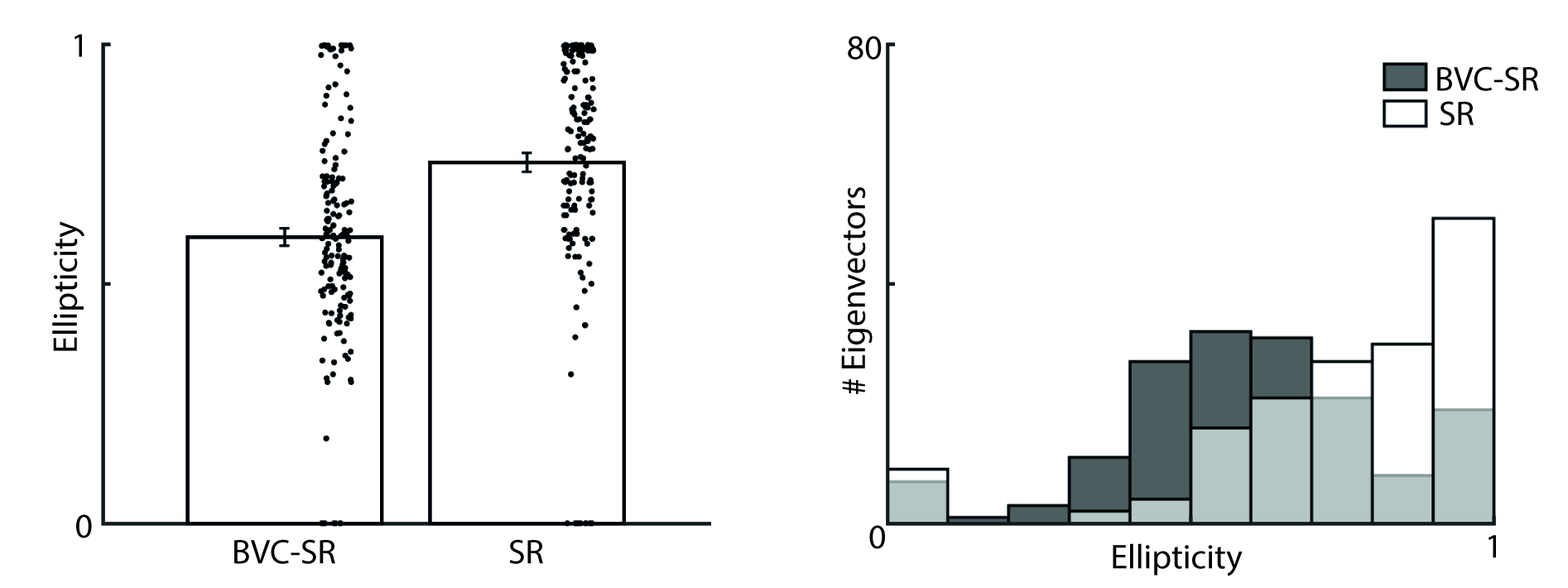

Supplement: Supplementary file 2 — Supplementary Figure 1 Grid fields generated using eigenvectors from the BVC‐SR model are less elliptic than those from the standard SR model. Lower values indicate more circular fields and larger values indicate more elliptic fields, with a value of 0 indicating a perfect circle. a) Grid fields generated using the BVC‐SR model had significantly lower ellipticity than the standard SR model (mean field ellipticity ± SD: 0.59 ± 0.23 vs. 0.75 ± 0.25; t[318] = −5.93; p < 0.001), and were similar to observations of real grid cells (Krupic et al., 2015). b) Histogram of the grid field ellipticity (N = 160 eigenvectors) [file HIPO-30-1347-s003.tif]

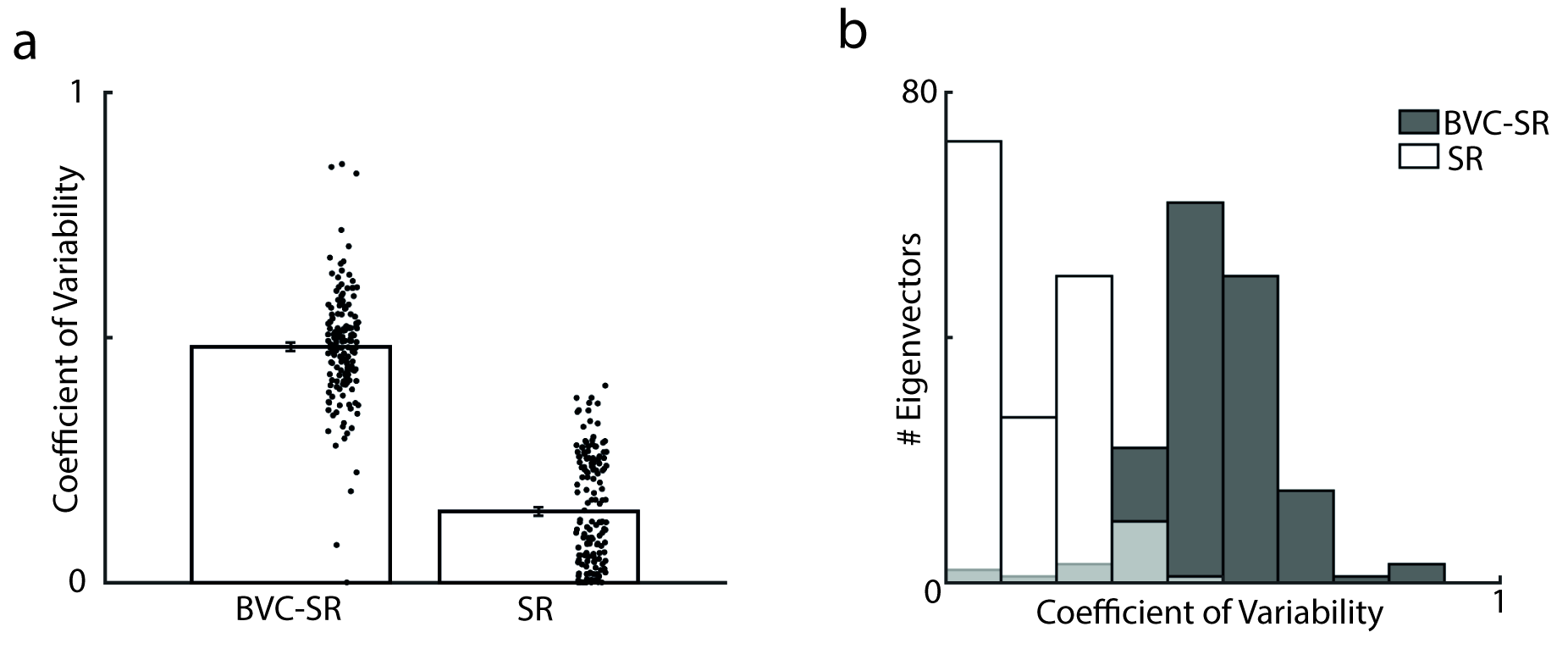

Supplement: Supplementary file 3 — Supplementary Figure 2 Grid fields generated using eigenvectors from the BVC‐SR model exhibit more firing rate variability than the standard SR model. Following the method of Ismakov et al., (2017), the peak firing rates of grid fields was used to compute a coefficient of variability for each eigenvector (CV; SD divided by mean). a) The CV for eigenvectors produced by the BVC‐SR model were significantly larger than that observed in the standard SR model (mean CV ± SD: 0.48 ± 0.11 vs 0.14 ± 0.11; t[318] = 26.5; p < 0.001), and similar to that observed in real grid cells (Ismakov et al., 2017). b) Histogram of the CV for each of the models (N = 160 eigenvectors). [file HIPO-30-1347-s001.tif]
